# Predictors for uptake of intermittent preventive treatment of malaria in pregnancy (IPTp) in Tanzania

**DOI:** 10.1186/s12889-015-1905-0

**Published:** 2015-06-07

**Authors:** Stephen M. Kibusi, Eunice Kimunai, Courtney S. Hines

**Affiliations:** College of Health Sciences, University of Dodoma, P.O. Box 259, Dodoma, Tanzania

**Keywords:** Malaria, Pregnancy, Intermittent preventive treatment of malaria in pregnancy, Sulfadoxine-pyrimethamine, Tanzania

## Abstract

**Background:**

Tanzania adopted Intermittent-preventive treatment of malaria in pregnancy (IPTp) policy in 2000; the guidelines at the time of the study recommended the timing of the first dose of intermittent preventive treatment in pregnancy (IPTp) with sulfadoxine-pyrimethamine (SP) (IPTp-SP) at 20–24 weeks and the timing of the second dose at 28–32 weeks. The aim of this study was to identify factors that are responsible for the uptake of IPTp among pregnant Tanzanian women. Further, this study aims to justify the need for appropriate interventions that would strengthen the Tanzanian IPTp program towards the realization of the Roll Back Malaria (RBM) targets.

**Methods:**

Data were analyzed from the 2011–2012 Tanzania HIV and Malaria Indicators Survey (THMIS) of 1,616 women aged 15–49 years who had a live birth in the 2 years prior to the survey and received antenatal care (ANC) services.

**Results:**

Logistic regression analysis results showed that (1) being in the age groups 30–34 and 35–39 versus other age groups and being married or living with partner versus those who reported as never married or divorced/separated were associated with high uptake of IPTp; (2) women pregnant with their first or second child versus those who already have had two or more children had higher odds of completing the recommended number of IPTp dosage; and (3) being a resident from the Eastern Zone versus Lake Zone as well as having the first antenatal visit in the first or second trimester versus third trimester were associated with higher uptake of IPTp.

**Conclusion:**

Applying these results could contribute to positive social change by helping providers, clinics, and organizations seeking to increase IPTp uptake among ANC attendees and providing health education programs to women, especially those residing in rural areas. This study could also help achieve United Nations Millennium Development Goals (MDG) 6 (combat HIV/AIDS, Malaria and Other Diseases).

## Background

Malaria, a mosquito-borne illness, which is preventable and treatable, is a major public health concern. In 2013, 97 countries had ongoing malaria transmission. An estimated 3.4 billion people are at risk for malaria. In 2012, there were an estimated 207 million cases of malaria and an estimated 627,000 deaths. 90 % of all malaria deaths occur in sub-Saharan Africa. In 2012, malaria killed an estimated 482,000 children under five years of age. That is 1,300 children every day, or one child almost every minute. Between 2000 and 2012, the increase in malaria interventions helped to reduce malaria incidence rates by 25 % globally, and by 31 % in the World Health Organization (WHO) African Region. Additionally, the global malaria mortality rate was reduced by 42 % during the same period, and the decrease in the malaria mortality rate in the WHO African Region was 49 %. During this same time period, increased malaria interventions saved an estimated 3.3 million lives. 90 %, or 3 million of these, are in the under-five age group in sub-Saharan Africa [1].

In Tanzania, the number of reported cases of malaria in 2010, 2011, and 2012 were 1,278,998; 2,150,761; and 1,986,955, respectively. The number of reported deaths from malaria in 2010, 2011, and 2012 were 15,867; 11,806; and 7,820 respectively [2]. Although the number of deaths from malaria has decreased (which is thought to be due to the increase in malaria interventions), Tanzania still has a high burden of malaria disease and each year many people become infected with the disease.

Malaria infection during pregnancy is also a major public health concern and poses many risks and threats to the mother, her unborn fetus, and the newborn. Maternal illness and low birth weight due to malaria are likely the result of *Plasmodium falciparum* infection, which occurs predominantly in Africa. Infection with *P. falciparum* can lead to chronic anemia, placental malaria infection, and low birth weight, all of which increase the risk of neonatal death [3]. Recommendations for the treatment and prevention of malaria during pregnancy include the following: (1) use of long-lasting insecticidal nets (LLINs); (2) intermittent preventive treatment in pregnancy (IPTp) with sulfadoxine-pyrimethamine (SP) in areas of stable malaria transmission of sub-Saharan Africa; and (3) prompt diagnosis and effective treatment of malaria infections [3].

Given the health risks that malaria infection during pregnancy poses to the mother, her unborn fetus, and the newborn, adequate measures should be taken to treat and prevent malaria infection during pregnancy. Intermittent preventive treatment of malaria in pregnancy (IPTp) is a full course of therapeutic antimalarial medicine given to pregnant women at routine prenatal visits. The antimalarial medicine is given to the mothers regardless of whether they are infected with malaria. IPTp helps to reduce maternal malaria episodes, maternal and fetal anemia, placental parasitemia, low birth weight, and neonatal mortality [4]. Current recommendations from WHO for IPTp include administration of sulfadoxine-pyrimethamine (SP) to pregnant women at each scheduled antenatal care (ANC) visit, starting as early as possible in the second trimester, provided that the doses are given at least one month apart. SP should not be given during the first trimester of pregnancy. WHO recommends a schedule of at least four ANC visits during pregnancy [5]. Tanzania’s current IPTp-SP guidelines are reflective of the current WHO recommendations.

Persistent, high levels of SP resistance markers have been identified in Tanzania by researchers who conducted a cross-sectional survey of six different regions in Tanzania [6]. Additionally, the rapid spread of SP resistance led Tanzania to replace SP therapy with artemisinin-based combinational therapy (ACT) in 2006 [7]. Due to safety concerns for ACT use during pregnancy, SP continues to be used in IPTp [6]. Given the importance of IPTp-SP in prevention of malaria in pregnancy and its resulting outcomes, such as maternal illness and low birth weight, WHO continues to recommend IPTp-SP use [4].

According to WHO, among the approximately 780 million persons at risk for contracting malaria in endemic countries in sub-Saharan Africa, an estimated 32 million pregnant women could benefit from IPTp each year. However, in the last few years, declining efforts to scale-up IPTp in several African countries has been observed [4]. Of importance is the fact that IPTp noticeably lags behind other malaria control measures in high-burden countries. Low levels of antenatal clinic attendance do not appear to be a contributing factor; however, uncertainty among health workers about SP administration may play a role [4].

Florey [8] analyzed demographic health surveys (DHS) in Sub-Saharan African countries in an effort to identify factors contributing to decreased IPTp delivery and found that IPTp was effectively delivered for only 18 % of targeted women. 83 % of women attended an antenatal clinic (ANC) at least once and 97 % of women who received one dose of SP attended an ANC twice. Therefore, access to ANC service was not found to be a major reason for the low rate of IPTp delivery. The study did, however, find that IPTp delivery is inadequate in these countries. Number and timing of ANC visits, type of health facility, and malaria transmission risk were identified as important predictors of IPTp delivery [8].

Another study found that in 31 countries with IPTp policies, 19 million pregnant women were unprotected by IPTp. This same study found that low coverage with intermittent preventive treatment contrasted with high ANC attendance which suggests that there are missed opportunities when women attend clinics but are not given intermittent preventive treatment [9]. Several other studies have investigated factors affecting uptake of and access to intermittent preventive treatment. These studies identified unclear messages about IPTp (specifically, timing of doses), limited supply of SP, limited understanding of IPTp, late enrollment or irregular ANC visits, and nurse underachievement as factors contributing to decreased uptake of intermittent preventive treatment [10-14].

The aim of this study is to identify the factors associated with the uptake of IPTp among pregnant women in Tanzania and to justify the need for appropriate interventions that will strengthen the Tanzania IPTp program towards the realization of the Roll Back Malaria (RBM) targets.

## Methods

### Design

Cross-sectional survey of nationally representative sample of individuals aged 15–49 years living in Tanzania in the period between December 2011 and May 2012.

### Data sources

The study was based on data from the 2011/12 Tanzania HIV and Malaria Indicators Survey (THMIS), conducted by the National Bureau of Statistics (NBS) in collaboration with the Tanzania Commission for AIDS (TACAIDS) and the Zanzibar AIDS Commission (ZAC), the Ministry of Health and Social Welfare (MoHSW) and the USAID-funded MEASURE DHS project from December 16, 2011, to May 24, 2012.

The sampling frame used for the 2011–12 THMIS was developed by the National Bureau of Statistics (NBS) after the 2002 Population and Housing Census (PHC) which covered all of the 30 regions of Tanzania. The sampling frame excluded nomadic and institutional populations such as persons in hotels, barracks, and prisons. The sample was selected in two stages. The first stage involved selecting sample points (clusters) consisting of enumeration areas (EAs) delineated for the 2002 PHC. A total of 583 clusters were selected. On the Mainland, 30 sample points were selected in Dar es Salaam and 20 were selected in each of the other 24 regions. In Zanzibar, 15 sample points were selected in each of the five regions. The second stage of selection involved the systemic sampling of households. A household listing operation was undertaken in all the selected areas prior to the fieldwork. From these lists, households to be included in the survey were selected. Approximately 18 households were selected from each sample point for a total sample size of 10,496 households. Weighting factors were added to the data file so that the results will be proportional at the national level. All women and men age 15–49 who were either permanent residents of the selected households or visitors who stayed in the household the night before the survey were eligible to be interviewed.

### Questionnaire

Two questionnaires were used for the 2011–12 THMIS: the Household Questionnaire and the Individual Questionnaire. These questionnaires are based on the MEASURE DHS standard AIDS Indicator Survey and Malaria Indicator Survey questionnaires and were adapted to reflect the population and health issues relevant to Tanzania. The questionnaires were translated into Kiswahili; the national language of Tanzania. Data presented in this study is based on the individual questionnaire.

Completed interviews were obtained for 10,967 women, yielding a response rate of 96 %. Of the 9,388 eligible men identified, 8,352 were successfully interviewed (89 % response rate). The principal reason for non-response among both eligible women and men was the failure to find them at home despite repeated visits to the households.

### Sample selection

Questions regarding malaria in pregnancy were administered to women who have had a pregnancy in the past 5 years before the survey. To control recall bias we selected 3,555 women with a live birth 2 years prior to the survey out of the 10,967 women who completed the interviews. IPTp uptake was verified on the antenatal card, therefore only women with a live birth in the past 2 years and who attended antenatal services were included.

**Fig. 1:**
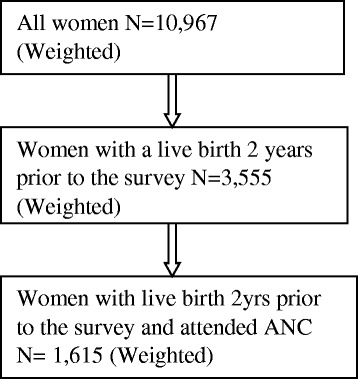
Sample selection

Out of the 3,555 women, 1,809 were excluded with 1,746 women remaining who had their antenatal cards at the time of survey as a proof of antenatal care attendance during pregnancy. Further, 130 women were excluded for lack of information about uptake of IPTp remaining with a sample of 1,616 women who were included in this study (Fig. 1).

### Variables

Guided by literature review, useful variables were identified from the 2011–12 THMIS Individual questionnaire. A conceptual framework was developed comprised of a list of primary independent variables (such as socio-economic and demographic variables), modifiable variable/intermediate independent variables, which are variables that could potentially be affected by the primary independent variables (such as number and timing of antenatal visits as well as birth under professional assistance) and lastly the outcome variable.

### Outcome measures

Uptake of IPTp was the outcome variable of interest defined as uptake of the recommended two or more doses of IPTp; one during the second trimester and the second during the third trimester (WHO, 2003). Women who did not take or took less than the recommended number of dosage were categorized as having low uptake of IPTp.

### Independent variables

This study included several socioeconomic and demographic variables that have been theoretically and empirically linked to malaria in pregnancy and uptake of IPTp. Participants’ age was categorized as follows: 15–24, 25–34, and 35–49 years of age. The participant’s area of residence was categorized as being rural or urban. The education level of women was categorized as follows; no education, primary incomplete, primary complete and secondary or higher education. We categorized a woman’s marital status into never married, separated/widowed and married. Occupation was categorized as unemployed, self employed and employed. Participant’s wealth index was categorized as follows: poorest, poorer, middle income, richer and richest. The wealth index is constructed using household asset data, including ownership of consumer items ranging from a television to a bicycle or car, as well as dwelling characteristics, such as source of drinking water, sanitation facilities, and type of flooring material. The wealth index was created in three steps. In the first step, a subset of indicators common to urban and rural areas was used to create wealth scores for households in both areas. In the second step, separate factor scores were produced for households in urban and rural areas using area-specific indicators. The third step combined the separate area-specific factor scores to produce a nationally applicable combined wealth index by adjusting area-specific scores through a regression on the common factor scores. The resulting combined wealth index has a mean of zero and a standard deviation of one. Wealth quintiles (from lowest to highest) were formed by assigning the household score to each participant, ranking each person in the study by that score, and then dividing the ranking into the five wealth index quintiles. We included parity defined as a woman’s number of live births that were categorized as: no child, 1 child, 2 children, and 3+ children. We further categorized Tanzania into nine zones, which tend to have different seasons and malaria prevalence. The zones included Eastern, Western, Southern, Southern High, South West Highlands, Central, Northern, Lake and the Zanzibar zone.

We also included intermediate factors potentially related to the uptake of IPTp and malaria in pregnancy. We categorized access to information about malaria whether a woman ever heard or never heard about IPTp (yes or no). We assessed access to antenatal services by including two variables; timing of the 1st Antenatal visit and the source of anti-malarias during the most recent pregnancy prior to the survey. Participant’s timing of the 1st Antenatal visit was categorized as being in; 1st trimester, 2nd trimester or 3rd trimester. Source of anti-malarias was categorized as antenatal centers or other sources.

### Statistical analyses

We calculated descriptive statistics for sample’s socio-demographic characteristics and IPTp uptake. Chi-square test was used to assess socio-demographic differences in IPTp uptake. The level of significance was set at *P* < 0.05 (2-tailed) for all the analyses. Both bivariate and multiple logistic regression were used to generate crude (OR) and adjusted odds ratios (AOR). Odds ratios were estimated to assess the strength of the associations and used the 95 % confidence intervals (CIs) for significance testing. All the covariates which showed statistically significant relationship (*P* < 0.05) with the outcome variable after Chi-square test were entered simultaneously into the multiple regression model. Analyses were performed using SPSS version 16. Sample weighting was applied to allow for adjustments for the cluster sampling design and sampling probabilities across clusters and strata.

### Ethical considerations

Data collection procedures for the THMIS were approved by Tanzania’s National Institute for Medical Research (NIMR), the Zanzibar Medical Ethics and Research Committee (ZAMREC), the Institutional Review Board of ICF International, and the Centers for Disease Control and Prevention in Atlanta. The protocol of the survey was reviewed and approved by the Tanzania’s National Institute for Medical Research (NIMR), the Zanzibar Medical Ethics and Research Committee (ZAMREC), the Institutional Review Board of ICF International, and the Centers for Disease Control and Prevention in Atlanta. All the participants were asked to provide verbal informed consent after being read a document emphasizing the voluntary nature of the survey. Interviews were conducted under the most private conditions afforded by the environments encountered. If privacy could not be ensured, the interviewers were instructed to skip the module.

## Results

Table 1 shows the distribution of participants by socio-demographic characteristics and uptake of IPTp. Most of the participants have completed at least primary education. There was a significant relationship (*p* < 0.001) between education level and uptake of IPTp with the highest percentage of those with no education (75 %) having reported low uptake. Participants in the age groups 30–34 and 35–39 years were proportionally more likely to complete uptake of IPTp than those in other age groups (uptake at 42 and 40 % respectively) (*p* < .01). Uptake was lower among participants who were self-employed (30.4 %) than other occupational groups (*p* < .001).

**Table 1:** The distribution of participants by socio-demographic characteristics and uptake of IPTp

Variables	% of women who took IPTp				*P*-value
	<2 doses		2+ doses		
	n	%	n	%	
Education					
No education	233	75.2	77	24.8	
Primary inco	127	68.3	59	31.7	***
Primary comp.	599	64.5	330	35.5	
Secondary+	104	54.7	86	45.3	
Age groups					
15–19	157	74.4	54	25.6	
20–24	294	68.5	135	31.5	
25–29	263	66.1	135	33.9	**
30–34	164	60.1	109	39.9	
35–39	127	57.7	93	42.3	
40–44	52	70.3	22	29.7	
45–49	7	63.6	4	36.4	
Occupation					
Unemployed	127	58.3	91	41.7	
Self employed	748	69.6	327	30.4	***
Employed	187	58.3	134	41.7	
Marital Status					
Never married	90	62.5	54	37.5	
Divorced/Separated	80	72.1	31	27.9	
Married	893	65.6	468	34.4	

Here *, ** and *** indicate *p* < 0.05, *p* < 0.01 and *p* < 0.001 respectively. *N* = 1616. Participants missing some information on independent variables: 2 on occupation

Table 2 shows the distribution of participants by potential factors which may affect uptake of IPTp. Distribution of participants by wealth, showed higher uptake of IPTp among the richest group (50.2) and much lower uptake among the poorest whom only 28 % reported to have completed the recommended 2+ doses (<0.001). Comparing participants’ uptake levels by reported number of children (parity); around 58 % of those with no children took the recommended number of dosages; the highest among all groups (*p* < 0.001). Surprisingly, mothers with three or more children had the lowest uptake (around 24 % only took the recommended number of dosages). Timing of the first visit to an antenatal clinic where IPTp services are provided had a significant relationship to completion of uptake of IPTp (*p* < 0.001) with participants making their first visit during the third trimester having the lowest level of uptake. Only 18 % of women making their first visit during the third trimester completed 2 or more doses as compared to 44 % of those who started their visit right on time in the first trimester.

**Table 2:** Distribution of participants by uptake of IPTp and the potential factors affecting uptake (Chi-Square)

Variable	% of women who took IPTp				*P*-value
	<2 doses		2+ doses		
	n	%	n	%	
Wealth Index					
Poorest	212	72.4	81	27.6	
Poorer	249	66.4	126	33.6	
Middle	233	71.7	92	28.3	***
Richer	223	68.0	105	32.0	
Richest	146	49.8	147	50.2	
Zones					
Eastern	99	44.8	122	55.2	***
Western	97	67.4	47	32.6	
Southern	34	57.6	25	42.4	
S Highlands	99	57.6	73	42.4	
SW Highlands	108	70.1	46	29.9	
Central	106	69.3	47	30.7	
Northern	74	59.2	51	40.8	
Lake	411	77.3	121	22.7	
Zanzibar	36	64.3	20	35.7	
Parity					
No child	11	42.3	15	57.7	
One child	315	58.9	220	41.1	***
Two Children	397	65.3	211	34.7	
3+ Children	340	76.4	105	23.6	
1st ANC Visit					***
1st trimester	149	55.8	118	44.2	
2nd trimester	733	65.1	393	34.9	
3rd trimester	182	81.6	41	18.4	
Ever heard about IPTp					
Yes	555	60.0	370	40.0	
No	508	73.5	183	26.5	***
Dose Source					
Antenatal visit	498	50.3	492	49.7	***
Other source	566	90.4	60	9.6	

Here *, ** and *** indicate *p* < 0.05, *p* < 0.01 and *p* < 0.001 respectively. *N* = 1616. Participants missing some information on independent variables: 2 on wealth & 2 on parity

There was significant variation in terms of uptake of IPTp by the region in Tanzania where a participant resides (*P* < 0.001). Proportionally, participants from the Eastern, Sothern and Southern highlands had higher uptake compared with regions like the Lake Zone where uptake was around 23 % only.

Awareness about malaria prevention programs was high and significantly related to uptake of IPTp (*p* < 0.001) with those reporting to have ever heard about IPTp recording higher uptake than those who didn’t (40 % versus 27 %). Source of anti-malarial medications during pregnancy was also significantly related to IPTp uptake (*p* < .001). Participants who received anti-malarial medications during antenatal visits reported higher uptake (50 %) compared with only 10 % among those who received the medications from other sources.

The results of logistic regression analysis presented in Table 3 below, indicate that being in the age groups 30–34 and 35–39 years, being married or living with a partner, were associated with higher uptake of IPTp controlling for other factors. Logistic regression analysis also showed that women at first or second pregnancy had higher odds of completing the recommended number of IPTp dosage than those who already have had two or more children. Being a resident from the Eastern Zone as well as having the first antenatal visit in the first or second trimester was associated with higher uptake of IPTp.

**Table 3:** Adjusted Odds Ratios (AOR) for factors associated with uptake of IPTp among women who attended ANC services and had a live birth 2 years before the survey (*N* = 1616)

Variable	AOR	95 % CI	P value
Age groups			
15–19	1		
20–24	1.28	0.83–1.98	
25–29	1.38	0.88–2.16	
30–34	1.88	1.16–6.05	**
35–39	2.25	1.36–3.64	***
40–44	1.17	0.60–2.29	
45–49	1.69	0.37–7.64	
Marital Status			
Married	1		
Never married	1.29	0.08–2.08	
Divorced/Separated	0.61	0.37–1.01	*
Zones			
Eastern	1		
Western	0.82	0.49–1.38	***
Southern	0.63	0.32–1.22	**
Southern Highlands	0.81	0.50–1.31	
Southern West Highlands	0.44	0.27–0.73	
Central	0.51	0.30–0.85	
Northern	0.68	0.41–1.13	
Lake	0.54	0.35–0.83	**
Zanzibar	0.46	0.23–0.93	*
Parity			
Three or more Children No child	1		
One child	3.08	1.21–7.83	*
Two Children	1.42	1.00–2.02	*
	1.10	0.80–1.51	
Timing of 1st ANC Visit:			
3rd trimester	1		
1st trimester	1.99	1.24–3.21	**
2nd trimester	1.94	1.29–2.90	***

Here *, ** and *** indicate *p* < 0.05, *p* < 0.01 and *p* < 0.001 respectively

## Discussion

The aim of this study was to identify factors that are responsible for the uptake of IPTp among pregnant Tanzanian women. Further, we hoped to justify the need for appropriate interventions that would strengthen Tanzanian IPTp programs towards the realization of the Roll Back Malaria (RBM) targets. The result of this study support the conceptual framework that guided this study, that is, uptake of IPTp is related to demographic factors (i.e., age, residence [rural/urban], education level, marital status, occupation, wealth index, parity, and geographical zones) and intermediate factors, which include access to information and access to services.

There was a significant relationship between uptake of IPTp and participants’ level of education, age, occupation, wealth, parity, and region of participants’ residence. Participants with no or low education level, young participants, self-employed participants, low-income participants, and participants with three or more children reported low uptake of IPTp. Uptake of IPTp was lowest in the Lake Zone region. There are several factors that contribute to the low uptake of IPTp among pregnant women. Researchers in Nigeria from Akinleye, Falade & Ajayi [15] found that IPTp use among pregnant women was very low and there was poor adherence despite utilization of Directly Observed Therapy (DOT). Based on our results we support the need for a concerted effort to increase awareness of IPTp among the public especially women of child bearing age [15]. Further, Ouma et al. [17] found that there was confusion about appropriate timing and lack of direct observation of IPTp in Kenya. Combined with our findings, we would support the need to train health workers on how to monitor IPTp adherence and also to communicate the importance of uptake of IPTp using simplified messages.

The timing of the first visit to an antenatal clinic where IPTp services are provided had a significant relationship to completion of uptake of IPTp with participants making their first visit during the third trimester having the lowest level of uptake. Tanzania’s national IPTp guidelines at the time of the study was that the timing of the first dose IPTp-SP was at 20–24 weeks and the timing of the second dose was at 28–32 weeks of pregnancy. This may lead to missed opportunities as most women attend ANC during or before the fourth month of gestation. In Tanzania, Anders, Marchant, Chambo, Mapunda & Reyburn [16] found that almost half the participants first attended ANC during or before the fourth month of gestation; however, 86 % of these early attendees did not receive IPTp on their first visit. Further, Anders et al. [16] found that in some facilities, SP was out of stock. We support the need to review national guidelines for when to start SP as efforts to encourage earlier attendance at ANC alone are unlikely to improve uptake of IPTp.

Awareness about malaria prevention programs was high and significantly related to uptake of IPTp with those reporting to have ever heard about IPTp recording higher uptake than those who did not. The source of anti-malarial medications during pregnancy also significantly related to IPTp uptake. Participants who received anti-malarial medications during antenatal visits reported higher uptake (50 %) compared with only 10 % among those who received the medications from other sources. In another Tanzanian study, Mubyazi, Bloch, Kamugisha, Kitua & Ijumba [14] found that pregnant women and ANC staff were generally aware of SP as the drug recommended for IPTp; however, some nurses and pregnant women expressed concerns about the safety of the drug during pregnancy. Further, Mubyazi [14] reported that compliance with taking SP was low especially when women were allowed to take the drug at home. Mubyazi [14] noted that compliance of direct observed therapy in administering SP for IPTp at ANC clinics was an issue due to shortage of clean water and cups. We support the need for proper planning of, support of, and training of health care workers and sustained sensitization of pregnant women at health facility and community levels about the benefits of IPTp for the women and their fetuses.

This study had the following limitations. First, the researchers only examined women who had a live birth in the past two years. This study did not include women whose children died before birth or were still born. Second, the primary source of the information collected from the research participants was self-report. Recall bias could influence the final reported information. Recall bias may result in underestimation or overestimation of past experiences or events. Third, we were limited on the variables that were available in the DHS questionnaire; thus, we could not explore other factors that could be relevant for this study, for example, beliefs/perception/attitude of the participants and their partners towards IPTp. Finally, the study did not address issues of stockpiling and availability of SP doses. There is a possibility that women who attended ANC per recommended guidelines failed to receive the medication because the medications were not available.

Recommendations for future research include qualitative studies to provide a much deeper understanding of the factors that contribute to uptake and adherence of IPTp. We recommend that future researchers explore issues of stock piling, provider’s beliefs, women and their partners’ beliefs, and the influence on women by their partners and the extended family at large related to uptake and adherence of IPTp.

## Conclusion

Tanzania adopted IPTp policy in 2000; the guidelines at the time of the study were that the timing of the first dose IPTp-SP was at 20–24 weeks and the timing of the second dose was at 28–32 weeks. Findings of this study could help providers, clinics, and organizations seeking to increase IPTp uptake among ANC attendees and providing health education programs to women especially those residing in rural areas. Applying these study results could also help achieve United Nations Millennium Development Goals (MDG) 6 (combat HIV/AIDS, Malaria and Other Diseases). We found that women who came late to ANC have low uptake of IPTp and this could indicate that the women who came late to ANC are also likely to miss other interventions embedded in ANC services, e.g. prevention of mother-to-child HIV transmission (PMTCT). This study also highlights the women who are at a high risk of missing IPTp services in Tanzania, e.g. women from the Lake Zone region and young women; therefore, future interventions should focus on these groups. Policy implications for this study include addressing low uptake of IPTp with the focus being on young mothers aged 15–19 years. Policy should also address the issue of women making their first ANC visit during the third trimester, thus contributing to low uptake of IPTp. Further, policy should support the linkage of IPTp and ANC because utilization of ANC increases uptake of IPTp.
